# Sedentary Behavior and Low Back Pain in Children and Adolescents: A Systematic Review and Meta-Analysis

**DOI:** 10.3390/healthcare14020233

**Published:** 2026-01-16

**Authors:** Inmaculada Calvo-Muñoz, José Manuel García-Moreno, Antonia Gómez-Conesa, José Antonio López-López

**Affiliations:** 1 Faculty of Physiotherapy, Occupational Therapy and Podiatry, UCAM Catholic University of Murcia, 30007 Murcia, Spain; 2 Faculty of Kinesiology, University of New Brunswick, Fredericton, NB E3B 5A3, Canada; josemanuel.garcia10@um.es; 3 Research Group Research Methods and Evaluation in Social Sciences, Mare Nostrum Campus of International Excellence, University of Murcia, 30100 Murcia, Spain; agomez@um.es; 4 Department of Basic Psychology and Methodology, University of Murcia, 30100 Murcia, Spain; josealopezlopez@um.es

**Keywords:** low back pain, children, adolescents, sedentary behavior, screen time, meta-analysis

## Abstract

**Highlights:**

**What are the main findings?**

- Dose–response meta-analysis shows a 26% increase in the odds of low back pain (LBP) for each additional hour of daily screen time in children and adolescents.
- Pairwise meta-analyses did not show a statistically significant association between screen time and LBP.

**What are the implications of the main findings?**

- Reducing screen-based sedentary behavior may help lower the risk of LBP in pediatric populations.
- High heterogeneity and risk of bias in existing studies highlight the need for standardized, high-quality research to better understand the relationship.

**Abstract:**

**Background/Objectives**: Low back pain (LBP) is increasingly prevalent among children and adolescents and represents a growing public health concern due to its potential persistence into adulthood. Screen-based sedentary behavior has substantially increased in pediatric populations. However, evidence regarding its association with LBP remains inconsistent, and the existence of a dose–response relationship is not well established. **Methods**: A systematic review and meta-analysis of observational studies was conducted in accordance with PRISMA guidelines. Studies examining the association between screen-based sedentary behavior and LBP in children and adolescents aged 6–18 years were included. Random-effects meta-analyses were used to pool continuous exposure estimates, and a multivariate random-effects dose–response meta-analysis was performed to assess changes in LBP risk across increasing levels of daily screen time. **Results**: A total of 30 studies were included. The pairwise meta-analysis of continuous exposure showed no statistically significant association between screen time and LBP, with OR = 1.02 (95% CI 0.65 to 1.59). In contrast, the dose–response meta-analysis demonstrated a significant positive association, with a 26% (95% CI 8% to 48%) increase in the odds of LBP for each additional hour of daily screen time. High between-study heterogeneity was observed, and most studies relied on self-reported measures of screen exposure and LBP, which may have introduced recall and misclassification bias and warrants cautious interpretation of the findings. **Conclusions**: Higher levels of screen-based sedentary behavior were associated with an increased risk of LBP in children and adolescents when examined using a dose–response approach, whereas pairwise meta-analyses did not identify a significant association. Nevertheless, substantial between-study heterogeneity and high risk of bias limit causal inference and require cautious interpretation.

## 1. Introduction

Low back pain (LBP) is one of the most common musculoskeletal complaints worldwide and is increasingly reported among children and adolescents. Although it was once considered rare in these age groups, studies have shown that LBP can affect between 12% and 50% of school-aged youth, depending on the criteria and methods used [1,2]. The presence of pain in early life is associated with a higher risk of persistence into adulthood, thereby contributing to the long-term global burden of disease [3].

In pediatric populations, LBP is usually nonspecific, meaning it cannot be attributed to a single pathological condition [4]. Its etiology is multifactorial, with physical inactivity, postural problems, psychosocial stressors, and sedentary lifestyles being recognized as important contributors [5]. Sedentary behavior, defined as any waking activity characterized by an energy expenditure ≤ 1.5 metabolic equivalents while sitting or reclining [6], has become a dominant lifestyle pattern in children and adolescents.

Screen-based sedentary time—including television, computers, smartphones, tablets, and video games—represents a large proportion of sedentary behavior in children and adolescents. Different screen types may impose varying postural demands; for example, television viewing is typically associated with more supported sitting, whereas smartphone and tablet use often involves sustained neck and trunk flexion, potentially altering spinal loading. Prolonged screen-based sitting has been associated with reduced activation of trunk stabilizing muscles, which may compromise spinal stability and increase susceptibility to mechanical stress and pain [7,8]. From a physiological perspective, “excessive” screen exposure does not imply a fixed threshold but rather prolonged and cumulative sitting durations that may exceed the spine’s adaptive capacity. Excessive screen time has also been associated with obesity, sleep problems, and psychological distress, which may further contribute to musculoskeletal pain [9].

Recent studies indicated that the COVID-19 pandemic markedly increased sedentary habits among children and adolescents. School closures, limited outdoor activities, and greater screen use for learning and leisure reduced physical activity levels, and were associated with poorer physical and mental health, including higher stress, worse sleep, and lower well-being [10].

Nevertheless, the evidence regarding the association between screen exposure and LBP in children and adolescents remains inconsistent. Some studies have reported significant positive associations [8,11], while others have failed to find clear evidence of such a link [5]. This heterogeneity likely reflects differences in study design, exposure and outcome measurement, and population characteristics.

As sedentary behavior and screen exposure continue to rise globally among children and adolescents, and their potential adverse effects on spinal health become more evident, it is imperative to conduct a meta-analysis to clarify the relationship between these public health concerns. Therefore, this meta-analysis aims to quantify the association between screen-based sedentary behavior and LBP in children and adolescents.

## 2. Materials and Methods

This meta-analysis was conducted following the Preferred Reporting Items for Systematic Reviews and Meta-Analyses (PRISMA) guidelines [12]; for more information, see Supplementary Table S1. Additionally, this study was registered with PROSPERO (CRD420251105673).

### 2.1. Eligibility Criteria

The eligibility criteria were defined a priori according to the PICOS framework. Eligible studies included children and adolescents aged 6 to 18 years, provided that participants did not present with specific spinal pathologies or other medical or traumatic conditions known to cause LBP. The exposure of interest was sedentary behavior measured as daily screen time, including the use of television, computers (desktop or laptop), tablets, smartphones, or gaming consoles, considering both leisure-related and educational contexts, but excluding screen time during school hours.

Given the multifactorial nature of LBP, we prioritized adjusted estimates where available. However, the degree and type of psychosocial adjustment varied across studies, and residual confounding cannot be ruled out.

The exposure of interest was restricted to leisure-time screen-based sedentary behavior. Educational screen use was excluded because it is largely compulsory, more standardized in terms of posture and duration, and less amenable to behavioral modification. In contrast, leisure-time screen use is typically discretionary, more heterogeneous in ergonomic conditions, and more likely to involve prolonged uninterrupted sitting, which may be more relevant to the development of LBP.

Comparators were groups with lower levels of screen time exposure or studies reporting categorical comparisons such as low versus high screen use; in the absence of a direct comparator group, studies reporting measures of association between screen time and LBP were also considered. The outcome of interest was LBP, either self-reported through validated questionnaires or surveys or clinically assessed. Studies were required to provide quantitative measures of association such as odds ratios, risk ratios, correlation coefficients, or prevalence estimates. Eligible study designs included observational studies, specifically cross-sectional, cohort, and case–control studies, and only articles published in peer-reviewed journals in English or Spanish were included.

Exclusion criteria encompassed studies addressing specific LBP with an identifiable cause, such as scoliosis, trauma, infection, tumours, or neurological disorders. In addition, studies were excluded if they did not provide explicit measurements of screen time or sedentary sitting, if they were clinical trials, qualitative studies, reviews, editorials, or case reports, if they were published in languages other than English or Spanish, or if they lacked sufficient quantitative data to calculate effect sizes.

### 2.2. Data Sources and Search

A comprehensive literature search was conducted from database inception to September 2025 in MEDLINE, Scopus, Web of Science, SciELO, PEDro, and LILACS. The search strategy combined Medical Subject Headings (MeSH) and free-text terms related to LBP, sedentary behavior, and pediatric populations. In addition, the reference lists of all included articles and relevant reviews were manually screened to identify further eligible studies. Language restrictions (English and Spanish) were applied during the database search stage. Grey literature was not included, which may increase the risk of publication bias; however, inclusion was restricted to peer-reviewed studies to ensure methodological rigor.

The search process was performed independently by two reviewers (ICM and JMGM), who systematically screened and retrieved the records. Subsequently, all authors reviewed the potentially eligible studies, discussed any discrepancies, and reached a consensus on the final list of included articles. The complete search strategy for each database is available in Supplementary Table S2.

### 2.3. Study Selection Process

After the removal of duplicates, the titles and abstracts of all retrieved records were independently screened by two reviewers (ICM and JMGM) to identify potentially relevant studies. Full texts of the selected articles were then assessed in detail to ensure compliance with the predefined inclusion and exclusion criteria. Disagreements were resolved through discussion, and when necessary, a third author was consulted for arbitration (AGC); however, this was not required.

### 2.4. Data Extraction Process

Data extraction from the included studies was carried out using a previously developed coding manual, which was based on Lipsey’s recommendations [13]. The variables have been grouped into three different categories: substantive (context and participant), methodological, and extrinsic variables. For more information about the coded variables, see Supplementary Table S3.

Data from each study were independently extracted by two reviewers (ICM and JMGM). Disagreements were resolved through discussion, and when necessary, a third author was consulted for arbitration (AGC). Inter-rater reliability was evaluated after the consensus process using Cohen’s kappa coefficient and the intraclass correlation coefficient (ICC) [14], both of which indicated perfect agreement (κ = 1.0; ICC = 1.0)

### 2.5. Assessment of Methodological Quality and Risk of Bias

The methodological quality and risk of bias (RoB) of the included studies were assessed using the ROBINS-E tool (Risk of Bias in Non-randomized Studies of Exposures, version 2022) [15], which is specifically designed for observational studies evaluating the impact of an exposure. Two reviewers (ICM and JMGM) independently conducted the risk of bias assessment. The ROBINS-E tool evaluates seven domains, including confounding, participant selection, classification of exposures, deviations from intended exposures, missing data, measurement of outcomes, and selection of the reported result. Each domain was rated according to the tool’s guidelines, and a final overall risk of bias judgment was assigned as low, some concerns, high, or very high.

Key confounders considered a priori included physical activity levels, body mass index, and sex. Studies with limited or no adjustment for these variables were generally rated as having a higher risk of bias due to confounding, in accordance with the ROBINS-E guidance.

Prior to formal assessment, reviewers underwent calibration exercises using sample studies. Risk of bias assessments were conducted independently by two reviewers. Inter-rater reliability was evaluated prior to consensus discussions using Cohen’s kappa coefficient. Disagreements were subsequently resolved through discussion, and when necessary, a third author was consulted for arbitration (AGC). The initial inter-rater agreement was excellent (κ = 1.0).

### 2.6. Statistical Analysis

The primary effect measure was the odds ratio (OR) for the association between screen-based sedentary behavior and LBP, extracting adjusted estimates in preference to unadjusted ones. We used two different statistical integration approaches depending on how this association was examined and reporting across studies. For both approaches, a log-linear relationship between screen time and LBP was assumed and restricted maximum likelihood was used for parameter estimation. We back-transformed results to the original ratio scale to facilitate interpretation. Formal non-linear dose–response analyses were not conducted due to the limited number of exposure categories and substantial heterogeneity across studies.

First, we took a standard univariate meta-analytic approach to combine results from studies reporting a logistic regression between screen time (continuous exposure) and LBP. We assumed a random-effects model to account for expected heterogeneity among studies [16], with the Hartung–Knapp adjustment applied to improve the precision of confidence intervals [17]. A forest plots with 95% confidence intervals was generated to represent both individual study effects and the pooled effect size. Prediction intervals were also included to reflect the expected range of true effects in future studies. Heterogeneity was further assessed using the Q statistic and the I2 index. We planned to assess publication bias visually with funnel plots and statistically with Egger’s test. Subgroup analyses were also planned according to adjusted vs. raw estimates and type of screen.

Second, to combine studies with multiple exposure groups (e.g., different levels of screen time), we used a multivariate random-effects model, allowing us to examine a potential dose–response relationship and to draw predictions for specific exposure values. We used a two-stage approach, with the method proposed by Greenland and Longnecker to define the covariance matrix, and the Q statistic and the I2 index to assess heterogeneity [18]. Subgroup analyses were planned according to adjusted vs. raw estimates and type of screen.

All analyses were conducted using R software (version 4.5.1) [19] with the metafor [20] and dosresmeta packages [21]. The PRISMA checklist was used to verify the completeness and transparency of the meta-analysis reporting [12].

## 3. Results

### 3.1. Study Selection

**Figure 1:**
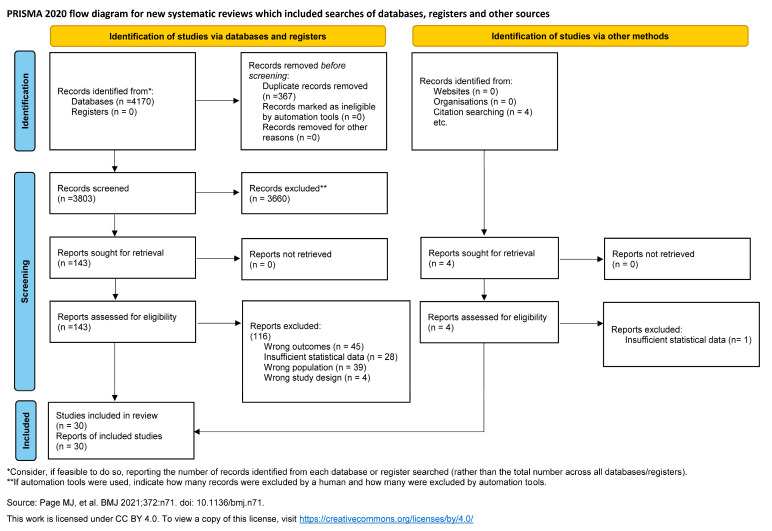
PRISMA flow diagram. Process of identification and selection of studies [12].

A total of 4170 articles were identified. After the elimination of duplicates, 3803 articles were retained for examination. Following the review of titles and abstracts, 147 articles were selected for full-text analysis to determine whether they met the inclusion criteria. Finally, 30 studies were included. The flow chart (Figure 1) provides a detailed description of the article selection process.

### 3.2. Study Characteristics

The 30 studies were published between 1988 and 2025 [22,23,24,25,26,27,28,29,30,31,32,33,34,35,36,37,38,39,40,41,42,43,44,45,46,47,48,49,50,51]. All studies were cross-sectional [22,23,24,25,26,27,28,29,30,31,32,33,34,35,36,37,38,39,40,41,42,43,44,46,47,48,49,50,51], except for one cohort study [45]. The studies were conducted in Switzerland [22,23,30], Norway [24], The Netherlands [25,49], Finland [26,29,32,36], Iran [27,37,43,51], Denmark [28,46], Slovenia [31], Brazil [33,35,40,44,45,47,48], Portugal [34,42], Japan [38], Tunisia [39], and Kuwait [41], and Jordan [50].

The participants were primarily recruited from educational institutions [22,23,24,25,27,28,30,31,32,33,34,35,36,37,39,40,41,42,43,44,45,47,48,51], with some studies including community-based recruitment [26,29,46,49,50] or sports organizations [36,38]. Of note, one study [36] used a mixed recruitment approach, including both educational institutions and sports organizations.

Most studies reported period prevalence of LBP, with recall periods ranging from 0.25 to 12 months [22,24,25,26,27,28,30,31,32,34,35,36,37,39,40,42,43,44,45,47,48,49,51], with 1-month and 12-month periods being the most common. A few studies assessed point prevalence [38,41,44,46], lifetime prevalence [23,29,33,44].

Screen exposure was assessed for television, computer, smartphone, tablet, and video games, either individually or in combination, with TVs and PCs being the most frequently studied devices [22,23,24,25,26,27,28,29,30,31,32,33,34,35,36,37,38,39,40,42,43,44,45,46,47,49,51]. For more information, see Table 1.

Variability in how screen exposure was categorized across studies may have attenuated associations in pairwise analyses. In contrast, dose–response approaches were better suited to capture cumulative exposure effects. Subgroup analyses by study period were not feasible due to limited reporting and overlap in exposure definitions. Given the predominance of cross-sectional designs, the findings reflect associations, and causality cannot be established; reverse causation cannot be ruled out.

#### 3.2.1. Sample

The studies included participants across different age groups, with most studies including mixed ages [22,23,27,30,31,33,34,35,37,38,41,42,47,48], several studies focusing solely on adolescents [24,25,26,28,29,32,36,39,40,44,45,49,51], and a few studies including only children [43,46,50]. The proportion of female participants ranged from 28.6% [38] to 74% [44], and in one study it was not reported [50].

#### 3.2.2. Screen-Based Sedentary Behavior Assessment

Regarding the assessment of personal data and LBP, the most common method was on-site questionnaires [22,23,24,25,27,28,30,31,32,33,34,36,37,39,40,41,43,44,45,47,48,49,51], followed by large-scale questionnaires [26,29,36,38,46,50], interviews [35,42,49], and parent reports [22,23]. For sedentary behavior, the most frequent method was also on-site questionnaires [22,23,24,25,27,28,30,31,32,33,34,36,39,40,41,43,44,45,47,48,49,51], followed by large-scale questionnaires [26,29,36,38,46,50], interviews [35,37,42,49], and parental reports [23]. Large-scale questionnaires generally included surveys distributed to participants at home or completed online. None of the included studies used objective measures such as accelerometers. Some studies combined more than one data-collection method for the same type of information to maximise response rates [22,23,36,49]. Most studies used the same data-collection method for personal/LBP variables and for sedentary behavior, although one study applied different approaches for these two types of information [37].

### 3.3. Risk of Bias Assessment

Most studies presented a high overall risk of bias [22,25,26,27,29,31,32,33,34,35,37,39,40,41,42,44,45,51], while four showed some concerns [28,30,43,47] and six were rated as low risk [23,24,36,38,46,48]. The domain most frequently rated as high risk was bias due to confounding, mainly because of limited adjustment for potential covariates. In contrast, most studies showed low risk in domains related to missing data, outcome measurement, and reporting bias. Two studies [49,50] were classified as very high risk, with several domains rated high. Detailed assessments are shown in Table 2.

### 3.4. Narrative Synthesis of Study Findings

Most studies reported category-based analyses [22,25,26,29,32,33,34,35,37,38,39,40,41,42,43,45,46,47,48,50], while some used continuous regression models [27,28,36,49], and a few applied ordinal regression [23,24,30]. A small number of studies did not directly report an association measure. In those cases, OR were calculated from raw data using two-by-two contingency tables [22,24,33,34,35,41]. The small number of studies using continuous exposure measures may have limited statistical power in pairwise analyses. More information is detailed in Table 3.

In general, most analyses were adjusted for potential confounders [23,24,26,28,29,30,32,36,38,39,40,43,45,46,47,48]. Almost all studies reported OR, with only one study presenting PR [40] and another reporting RR [46]. Regarding adjustment variables, each study reported a different model, primarily based on the authors’ preferences. Common covariates included age, sex, and BMI, although the specific selection varied substantially across studies, leading to considerable variability in the degree of adjustment.

Regarding sedentary behavior categories, there was substantial heterogeneity across studies. Reported categories ranged from very low exposure levels, such as 0.14–0.28 h/day [37,39], to very high levels, such as ≥7.60 h/day [47]. A temporal pattern was also observed. Earlier studies tended to define narrower exposure ranges and even included categories such as 0 h/day of TV viewing [22,23], whereas more recent studies reported higher minimum thresholds, such as <2 h/day [48], <3 h/day [50], or <3.25 h/day [47].

In addition, the types of screen-based devices assessed evolved over time. The earliest study that included TV exposure was published in 1988 [22]. PC use was first incorporated in 2004 [24], smartphone use in 2006 [26], video game use in 2014 [33], and tablet use in 2016 [36]. This progression reflects changes in technology availability and children’s screen-related habits over the decades.

There was substantial heterogeneity in the findings. Most studies identified a positive association between sedentary behavior and LBP in at least one of their analyses [22,26,28,29,32,36,38,39,40,41,43,45,46,47,50]. Others found no association in any of the analyses [23,24,25,30,33,34,35,37,48,49], and three studies reported sedentary behavior as a potential protective factor in some of their estimates [27,30,42]. The magnitude of the reported associations varied widely across studies, with OR values ranging from very small to large effects. In most studies, the strength of the association varied across exposure categories or analytical models, which further contributed to the variability in the findings.

### 3.5. Univariate Meta-Analysis

**Figure 2:**
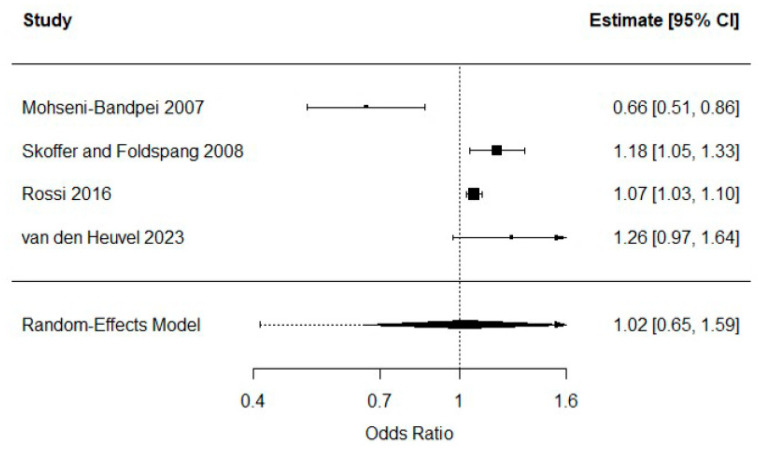
Random-effects meta-analysis of continuous screen time and LBP [27,28,36,49].

Only four studies reported logistic regression analyses in the required format to be included in the standard meta-analysis [27,28,36,49]. A random-effects model yielded an overall estimate of OR = 1.02 (95% CI 0.65 to 1.59), suggesting no evidence of an association between screen time (continuous exposure) and LBP. As Figure 2 shows, there was substantial heterogeneity among studies, and this was also reflected by the Q statistic (Q3 = 17.3, *p* = 0.0006). Furthermore, the I2 index revealed that 93.5% of the total variability was due to heterogeneity, and the 95% prediction interval provided further evidence of heterogeneity range of values between 0.41 and 2.54.

One of the studies reported an inverse association between screen time and LBP [27]. This study differed from the rest because the exposure and outcome windows were not aligned, as screen time was measured at a single time point while LBP referred to a one-month prevalence. The screen-time variable did not represent sitting since more than half of the sample reported watching TV while lying down. The estimate was unadjusted, and among the four studies with extractable data only two provided adjusted estimates; the two unadjusted studies were this one [27], which showed a reversed association, and another that showed a non-significant association. The exposure was not clearly defined in hours per day. This study also reported PC use, for which the OR was non-significant. Finally, the sample included both children and adolescents whereas the other studies included only adolescents. A sensitivity analysis excluding this study resulted in an overall OR = 1.12 (95% CI 0.81 to 1.54), which again provided no evidence of an association. Due to the small number of studies, no further analyses were conducted. The stronger association observed in adjusted analyses may reflect negative confounding, whereby factors such as physical activity or BMI masked the crude association

### 3.6. Dose–Response Meta-Analysis

**Figure 3:**
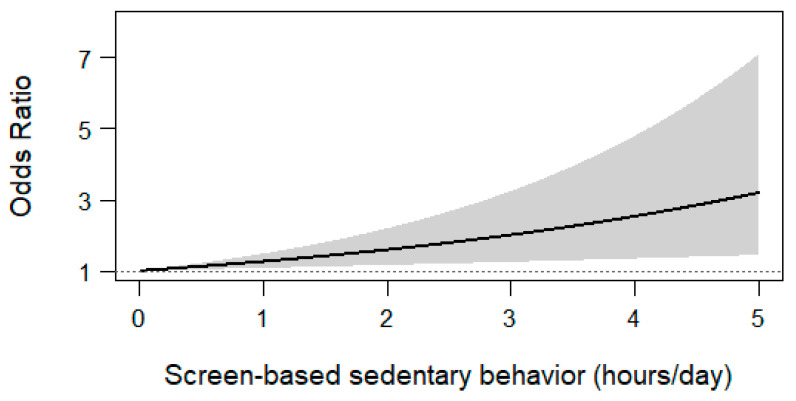
Screen-based sedentary behavior.

A total of 19 studies examined the association between screen time and LBP defining categories based on exposure levels [22,24,25,26,29,32,33,34,35,37,38,39,41,42,43,45,47,48,50]. Most of these studies reported multiple exposure groups, and hence a multivariate meta-analytic model was fitted to examine a potential dose–response relationship. Six studies that did not report cases and total group size could not be included in the analysis [26,29,32,37,47,50]. The analysis based on the remaining 13 studies [22,24,25,33,34,35,38,39,41,42,43,45,48] provided evidence of a significant log-linear dose–response association between screen time and LBP ($\hat{\beta }$ = 0.23, 95% CI 0.07 to 0.39). The increase in the odds of LBP for every additional hour of screen time was estimated at 26% (95% CI 8% to 48%). Nonetheless, the Q statistic showed evidence of heterogeneity (Q12 = 157.6, *p* < 0.0001) and the I2 index suggested that 92.4% of the observed variability was attributable to heterogeneity. Figure 3 shows the dose–response curve of the main analysis based on the 13 included studies, illustrating an increasing trend in the odds of LBP as screen time (dose) increases, with widening 95% CI at higher exposure levels.

The overall association was stronger for the four studies reporting adjusted estimates [38,39,43,48], which yielded a predicted increase in the odds of LBP of 57% (−3% to 153%) for every additional hour of screen time per day. On the other hand, the overall prediction for the nine studies reporting raw estimates [22,24,25,33,34,35,41,42,45] was 17% (2% to 33%).

With regard to screen type, TV was considered in nine studies [22,25,34,35,38,39,42,43,45], with an overall prediction of a 28% increase (95% CI 1% to 63%) in the odds of LBP for every additional hour of exposure. Combined exposure to TV and PC was considered in two studies [24,33], with an average prediction of 29% (7% to 56%). Last, two studies examined smartphone use [41,48], with an average prediction of 24% (12% to 37%). Therefore, there was little evidence of effect modification according to screen type.

## 4. Discussion

The aim of this study was to quantify the association between screen-based sedentary behavior and LBP in children and adolescents. Although the relationship between sedentary behavior and LBP has been previously examined in meta-analyses [52,53,54], a focused analysis was needed to specifically assess the role of screen-based sedentary behavior. By isolating sedentary time attributable to screen use, our findings may better inform the development of targeted interventions aimed exclusively at this modifiable behavior. Moreover, the dose–response analysis reported in the present study provided additional value.

One previous meta-analysis examined the association between sedentary behavior and LBP, as well as between screen use and LBP, in adults and in children and adolescents [52]. In children and adolescents, that meta-analysis reported an association between prolonged sedentary behavior and LBP of OR = 1.41 (95% CI 1.24 to 1.60), prolonged TV viewing and LBP of OR = 1.23 (95% CI 1.08 to 1.41), and prolonged use of computers, smartphones, or video games and LBP of OR = 1.63 (95% CI 1.36 to 1.95). However, the authors did not specify the exposure thresholds used or the reference groups applied in the pooled analyses. Notably, some of the studies included in that meta-analysis were also included in the present study.

A recent meta-analysis focused on overall sedentary behavior [53], rather than screen-based exposure, and examined both overall spinal pain and LBP. Using cross-sectional studies, the authors reported a small positive association between sedentary behavior and LBP of OR = 1.20 (95% CI 1.08 to 1.34), based on 17 studies reporting adjusted estimates. This work provides valuable evidence regarding the direction and magnitude of the association at a population level. Nonetheless, although longitudinal analyses were conducted for spinal pain overall, no causal inferences could be drawn specifically for LBP. Moreover, the cross-sectional LBP analysis relied on study-specific categorical exposure definitions, which limited comparability across studies and precluded the characterization of how risk changes across increasing levels of sedentary behavior or the quantification of a dose–response relationship.

Similarly, another meta-analysis [54] examined the association between screen-based sedentary behavior and LBP and reported a positive association for daily computer use of OR = 1.32 (95% CI 1.05 to 1.60), mobile phone use of OR = 1.32 (95% CI 1.00 to 1.64), and TV viewing of OR = 1.07 (95% CI 1.04 to 1.09). However, the pooled estimates were based on heterogeneous highest versus lowest exposure categories, limiting comparability across studies, and multiple estimates derived from the same studies were included, potentially leading to overlapping data. Notably, that study also conducted a dose–response meta-analysis based on seven studies (n = 57,831), reporting a linear increase of 8.2% in LBP risk per additional hour of daily computer use, with results primarily presented as percentage changes rather than underlying regression coefficients. In contrast, our dose–response meta-analysis included a larger number of studies, examined screen-based sedentary behavior more comprehensively, and reported a larger increase in the odds of LBP per additional hour of screen, estimated at 26% (95% CI 8% to 48%). This difference in magnitude may reflect broader exposure definitions, whereby the cumulative burden of multiple screen-based behavior exerts a stronger association with LBP than computer use alone, as well as differences in study selection and analytical approaches.

Our study did not identify a statistically significant association between LBP and screen-based sedentary behavior in the continuous analysis, whereas several previous studies reported significant associations using categorical exposure comparisons. This discrepancy may be partly explained by the conservative analytical approach adopted in the present meta-analysis. Specifically, studies using heterogeneous categorical definitions of screen time were not combined, as direct comparisons across incompatible exposure thresholds were avoided. Consequently, a categorical meta-analysis was not undertaken, and the quantitative synthesis was restricted to studies providing compatible continuous exposure data. In addition, when individual studies reported multiple screen-based exposures, a predefined hierarchy was applied, prioritising TV viewing, to ensure that only one estimate per study contributed to the pooled analysis. This strategy reduced the risk of overlapping data and overrepresentation of individual samples, but may also have attenuated pooled effect estimates compared with analyses that include multiple correlated exposures from the same study.

Several mechanisms may help explain the association between screen-based sedentary behavior and LBP in children and adolescents. Prolonged screen use often involves sustained sitting, non-neutral spinal postures, and limited activation of trunk muscles, which may increase mechanical load on the lumbar spine. These effects may be more pronounced during smartphone and tablet use, where unsupported and asymmetrical postures are common. In addition, excessive screen time has been associated with higher stress levels, sleep disturbances, and poorer mental health, factors that have also been linked to musculoskeletal pain. Together, these biomechanical, postural, and psychosocial factors may interact and contribute to the development or persistence of LBP in pediatric populations.

Importantly, the dose–response meta-analysis conducted in this study adds value by helping to interpret the substantial heterogeneity observed across the literature. Although some organisations propose daily screen time thresholds (e.g., 2 h per day) [55], there are currently no internationally accepted standards defining excessive or healthy levels of screen-based sedentary behavior. As a result, exposure categories continue to be defined according to author-specific criteria, which contributes to between-study heterogeneity and limits the interpretability of categorical pooled estimates.

Heterogeneity played a major role in the present findings. The included studies differed substantially in methodology, particularly in how LBP was defined and assessed and how sedentary behavior was measured (e.g., interviews, self-reported questionnaires, or parent-reported measures). Additional heterogeneity arose from differences in study populations, including wide age ranges encompassing both children and adolescents. Variability in exposure thresholds, together with differences in covariate adjustment across studies, further contributed to the high heterogeneity observed. The heterogeneity identified in this and previously discussed studies highlights the urgent need for standardization in this field. Harmonized definitions of LBP, consistent screen-based sedentary behavior thresholds, and agreed methodological approaches would improve comparability across studies. Where feasible, the use of clinical or hospital records to ascertain LBP may provide more robust and objective outcome definitions than self-reported measures, particularly for clinically relevant pain. While a leisure-time screen use threshold of 2 h per day is commonly proposed as a reference [55], internationally accepted standards are still lacking. Finally, there is a clear need for greater use and standardization of objective measures of sedentary behavior, such as accelerometers, which can provide more reliable and detailed information than self-reported measures.

### 4.1. Risk of Bias

Most included studies exhibited methodological limitations, primarily related to inadequate control of confounding, reflecting the lack of consensus on which variables are most relevant in this research area. At a minimum, future studies should account for key confounders such as biological sex, age, body mass index, physical activity, and socioeconomic status. In addition, the use of explicit causal frameworks, such as Directed Acyclic Graphs, may help guide appropriate confounder selection and reduce the risk of residual confounding, overadjustment, and collider bias, even in cross-sectional analyses [56,57].

Furthermore, screen time and LBP were predominantly assessed using self-reported measures, which may have introduced recall and misclassification bias. The use of objective measures, such as device-based assessments of screen exposure or clinical and hospital records for LBP, could help mitigate these sources of bias in future research.

### 4.2. Strengths and Limitations

The main strengths of this study include its specific focus on screen-based sedentary behavior and the use of a multivariate dose–response meta-analysis, which allowed the inclusion of multiple exposure categories and the modelling of risk across increasing levels of screen time. This approach provides more informative and policy-relevant estimates than traditional pairwise meta-analyses based on highest versus lowest exposure categories. In addition, conservative analytical choices were applied to strengthen the robustness of the findings and reduce the risk of bias and effect overestimation. These included the use of random-effects models, the avoidance of pooling studies with incompatible exposure definitions, and the prioritization of adjusted estimates.

Several limitations should be considered. First, all included studies relied on self-reported measures of both screen exposure and LBP, which are prone to recall bias, particularly in pediatric populations. Recall periods varied from a few weeks to 12 months, potentially introducing additional measurement uncertainty. Importantly, none of the studies used device-based assessments of screen time. Second, substantial heterogeneity across studies limited comparability and reduced the number of studies that could be pooled in meta-analyses. Many studies were assessed as having a high risk of bias. Furthermore, only one study used a longitudinal design, limiting the ability to examine changes in risk over time and to assess causal pathways. Consequently, the findings should be interpreted with caution. Third, reverse causality should be considered, as children and adolescents experiencing LBP may reduce participation in physical activities and preferentially engage in screen-based sedentary behaviors, either as a coping strategy or due to pain-related functional limitations. Given that most included studies were cross-sectional, the temporal direction of associations cannot be determined. Finally, changes in screen use posture over time, from predominantly TV viewing to handheld device use involving sustained flexed postures, may affect spinal loading differently. Additionally, data from younger children and adolescents were combined despite developmental differences in spinal growth and biomechanics, which may limit the generalizability of the findings.

### 4.3. Implications for Practice and Public Health

The findings support current recommendations to limit prolonged screen-based sedentary behavior in children and adolescents. Although the observed associations were modest, the widespread and increasing use of screens suggests that even small increases in risk may be meaningful at the population level. Clinicians should consider screen time as part of a broader lifestyle assessment when managing pediatric patients with LBP. From a public health and research perspective, future longitudinal and interventional studies are needed to determine whether reducing screen exposure leads to meaningful improvements in spinal health. Such studies should prioritize standardized definitions of screen-based sedentary behavior and LBP, use objective or validated measurement approaches where possible, apply explicit strategies to minimize bias, and adopt harmonized analytical frameworks to improve comparability across studies and reduce between-study heterogeneity. Future longitudinal and interventional studies using standardized, objective measures and improved methodological rigor are needed to clarify causality and inform prevention strategies.

## 5. Conclusions

This systematic review and meta-analysis suggest that higher levels of screen-based sedentary behavior are associated with an increased risk of LBP in children and adolescents when examined using a dose–response approach. While pairwise meta-analyses did not identify a significant association, the dose–response analysis indicated a progressive increase in risk with greater daily screen time, suggesting that screen exposure may act as a cumulative and potentially modifiable lifestyle factor. Nevertheless, substantial between-study heterogeneity and the high risk of bias across included studies limit causal interpretation and warrant cautious interpretation of the findings.

## Figures and Tables

**Table 1:** Characteristics of the studies.

Study	Study Design	Country	Age Category	% Female	Recruitment Setting	Type of Prevalence	Period Prevalence (Months)	Type of Screen Exposure
Balague et al. 1988 [22]	Cross-sectional	Switzerland	Mixed	51%	Educational institution	Period	0.25	TV
Balagué et al. 1994 [23]	Cross-sectional	Switzerland	Mixed	50.6%	Educational institution	Lifetime	NA	TV
Sjolie 2004 [24]	Cross-sectional	Norway	Adolescent	43%	Educational institution	Period	12	TV PC
Diepenmaat et al. 2006 [25]	Cross-sectional	Netherlands	Adolescent	50.5%	Educational institution	Period	1	TV PC
Hakala et al. 2006 [26]	Cross-sectional	Finland	Adolescent	55.6%	Community	Period	6	TV PC Smartphone
Mohseni-Bandpei et al. 2007 [27]	Cross-sectional	Iran	Mixed	52.3%	Educational institution	Period	1	TV PC
Skoffer and Foldspang 2008 [28]	Cross-sectional	Denmark	Adolescent	46.7%	Educational institution	Period	3	TV
Hakala et al. 2010 [29]	Cross-sectional	Finland	Adolescent	55.3%	Community	Lifetime	NA	PC
Erne and Elfering 2011 [30]	Cross-sectional	Switzerland	Mixed	55%	Educational institution	Period	1	TV Video Computer
Turk et al. 2011 [31]	Cross-sectional	Slovenia	Mixed	49.5%	Educational institution	Period	3	PC TV
Hakala et al. 2012 [32]	Cross-sectional	Finland	Adolescent	53.7%	Educational institution	Period	6	PC
Graup et al. 2014 [33]	Cross-sectional	Brazil	Mixed	50.9%	Educational institution	Lifetime	NA	TV Video games PC
Minghelli et al. 2014 [34]	Cross-sectional	Portugal	Mixed	54.8%	Educational institution	Period	12	TV Computer
Fernandes et al. 2015 [35]	Cross-sectional	Brazil	Mixed	48.4%	Educational institution	Period	12	TV PC
Rossi et al. 2016 [36]	Cross-sectional	Finland	Adolescent	52.8%	Educational institution + Sports organization	Period	3	TV PC Video games Mobile phone Tablet
Dianat et al. 2017 [37]	Cross-sectional	Iran	Mixed	53.4%	Educational institution	Period	1	PC Video games TV
Yabe et al. 2018 [38]	Cross-sectional	Japan	Mixed	28.6%	Sports organization	Point	NA	TV Video games
Ben Ayed et al. 2019 [39]	Cross-sectional	Tunisia	Adolescent	59.7%	Educational institution	Period	3	TV PC Video games
Bento et al. 2020 [40]	Cross-sectional	Brazil	Adolescent	51%	Educational institution	Period	12	TV PC Tablet Smartphone
Buabbas et al. 2020 [41]	Cross-sectional	Kuwait	Mixed	53.6%	Educational institution	Point	NA	Smartphone Tablets
Minghelli 2020 [42]	Cross-sectional	Portugal	Mixed	52.6%	Educational institution	Period	12	TV Mobile phone Video games
Rezapur-Shahkolai et al. 2020 [43]	Cross-sectional	Iran	Children	54.1%	Educational institution	Period	1	TV Smartphone Tablet PC Video games
Schwertner et al. 2020 [44]	Cross-sectional	Brazil	Adolescent	74%	Educational institution	Period, point, and lifetime	3	PC TV
de Vitta et al. 2021 [45]	Cohort	Brazil	Adolescent	43.8%	Educational institution	Period	12	TV PC Smartphone Tablet
Joergensen et al. 2021 [46]	Cross-sectional	Denmark	Children	52.3%	Community	Point	NA	TV PC
da Costa et al. 2022 [47]	Cross-sectional	Brazil	Mixed	55.1%	Educational institution	Period	0.25	TV Video games PC Smartphone
Lemes et al. 2022 [48]	Cross-sectional	Brazil	Mixed	55.4%	Educational institution	Period	0.25	Smartphone
van den Heuvel et al. 2023 [49]	Cross-sectional	Netherlands	Adolescent	52%	Community	Period	1.5	TV Video games
Obeidat and AL-Shalabi 2025 [50]	Cross-sectional	Jordan	Children	NI	Community	NI	NA	Smartphone
Rahmani et al. 2025 [51]	Cross-sectional	Iran	Adolescent	50%	Educational institution	Period	3	TV PC

NI: no information; NA: not applicable TV: television; PC: computer; Children: participants aged < 12 years; Adolescents: participants aged 12–18 years; Mixed: both children and adolescents.

**Table 2:** Risk of bias assessment.

Study	R-E1	R-E2	R-E3	R-E4	R-E5	R-E6	R-E7	R-Overall
Balague et al. 1988 [22]	High	Low	Low	Low	Low	Low	Low	High
Balagué et al. 1994 [23]	Low	Low	Low	Low	Low	Low	Low	Low
Sjolie 2004 [24]	Low	Low	Low	Low	Low	Low	Low	Low
Diepenmaat et al. 2006 [25]	High	Low	Low	Low	Low	Low	Low	High
Hakala et al. 2006 [26]	High	Low	Low	Low	Low	Low	Low	High
Mohseni-Bandpei et al. 2007 [27]	High	Low	Low	Low	Low	Low	Some concerns	High
Skoffer and Foldspang 2008 [28]	Low	Some concerns	Low	Low	Low	Low	Low	Some concerns
Hakala et al. 2010 [29]	Some concerns	Some concerns	Low	Low	Low	Low	Low	High
Erne and Elfering 2011 [30]	Low	Some concerns	Low	Low	Low	Low	Low	Some concerns
Turk et al. 2011 [31]	High	Low	Low	Low	Low	Low	Low	High
Hakala et al. 2012 [32]	High	Low	Low	Low	Low	Low	Low	High
Graup et al. 2014 [33]	High	Low	Low	Low	Low	Low	Low	High
Minghelli et al. 2014 [34]	High	Low	Low	Low	Low	Low	Low	High
Fernandes et al. 2015 [35]	High	Low	Low	Low	Low	Low	Low	High
Rossi et al. 2016 [36]	Low	Low	Low	Low	Low	Low	Low	Low
Dianat et al. 2017 [37]	High	Some concerns	Low	Low	Low	Low	Low	High
Yabe et al. 2018 [38]	Low	Low	Low	Low	Low	Low	Low	Low
Ben Ayed et al. 2019 [39]	Some concerns	Some concerns	Low	Low	Low	Low	Low	High
Bento et al. 2020 [40]	High	Some concerns	Low	Low	Low	Low	Low	High
Buabbas et al. 2020 [41]	High	Low	Low	Low	Low	Low	Low	High
Minghelli 2020 [42]	Some concerns	Low	Low	Low	Low	Low	High	High
Rezapur-Shahkolai et al. 2020 [43]	Some concerns	Low	Low	Low	Low	Low	Low	Some concerns
Schwertner et al. 2020 [44]	High	Some concerns	Low	Low	Low	Low	Some concerns	High
de Vitta et al. 2021 [45]	High	Low	Low	Low	Low	Low	Low	High
Joergensen et al. 2021 [46]	Low	Low	Low	Low	Low	Low	Low	Low
da Costa et al. 2022 [47]	Low	Some concerns	Low	Low	Low	Low	Low	Some concerns
Lemes et al. 2022 [48]	Low	Low	Low	Low	Low	Low	Low	Low
van den Heuvel et al. 2023 [49]	High	Low	Low	Low	High	Low	Low	Very high
Obeidat and AL-Shalabi 2025 [50]	High	Low	High	Low	High	Low	Low	Very high
Rahmani et al. 2025 [51]	High	Low	Low	Low	Low	Low	Low	High

**Table 3:** Associations between screen exposure and low back pain.

Study	Exposure Variable and Categories	Type of Analysis	Effect Measure (95% CI)
Balague et al. 1988 [22]	TV: 0–<1 h/d, 1–2 h/d, >2 h/d (vs. 0 h/d [Reference])	N/A	OR 0–<1 h/d: 0.89 (0.45–1.74); 1–2 h/d: 1.66 (0.86–3.22); >2 h/d: 3.05 (1.49–6.28) *
Balagué et al. 1994 [23]	TV: 0 h/d, <1 h/d, 1–2 h/d, >3 h/d	Multivariate ordinal logistic regression	OR 1.23 (1.0–1.52) ^a^
Sjolie 2004 [24]	TV and PC: 0.3–1.4 h/d, 1.6–2.1 h/d, 2.3–2.9 h/d, and 3–6.4 h/d (vs. 0.3–1.4 h/d [Reference])	Multivariate ordinal logistic regression	OR 1.70 (1.20–2.50) ^b^
		N/A	OR 1.6–2.1 h/d: 1.09 (0.36–3.29); 2.3–2.9 h/d: 2.54 (0.67–9.65); 3–6.4 h/d: 4.33 (1.21–15.44) *
Diepenmaat et al. 2006 [25]	TV: 1.51–2.5 h/d, 2.51–4 h/d, ≥4 h/d (vs. 0–1.5 h/d [Reference])	Bivariate logistic regression	OR 1.51–2.5 h/d: 0.60 (0.40–0.90); 2.51–4 h/d: 0.8 (0.60–1.10); ≥4 h/d: 0.8 (0.60–1.20)
	PC: 0.51–1.5 h/d, 1.51–3 h/d, ≥3.01 h/d (vs. 0–0.5 h/d [Reference])	Bivariate logistic regression	OR 0.51–1.5 h/d: 0.80 (0.60–1.20); 1.51–3 h/d: 0.90 (0.70–1.30); ≥3.01 h/d: 0.90 (0.60–1.30)
Hakala et al. 2006 [26]	TV, video and DVD: ≤1 h/d, 2–3 h/d, 4–5 h/d, and >5 h/d (vs. 0 h/d [Reference])	Multivariate logistic regression	OR ≤ 1 h/d: 0.90 (0.60–1.20); 2–3 h/d: 0.90 (0.70–1.20); 4–5 h/d: 1.00 (0.70–1.40); >5 h/d: 1.30 (0.70–2.30) ^c^
	Smartphone: ≤1 h/d, 2–3 h/d, 4–5 h/d, and >5 h/d (vs. 0 h/d [Reference])	Multivariate logistic regression	OR ≤ 1 h/d: 0.90 (0.70–1.10); 2–3 h/d: 1.00 (0.70–1.50); 4–5 h/d: 1.20 (0.50–2.60); >5 h/d: 1.00 (0.50–2.30) ^c^
	PC: ≤1 h/d, 2–3 h/d, 4–5 h/d, and >5 h/d (vs. 0 h/d [Reference])	Multivariate logistic regression	OR ≤ 1 h/d: 0.90 (0.70–1.20); 2–3 h/d: 0.90 (0.60–1.30); 4–5 h/d: 0.70 (0.30–1.60); >5 h/d: 2.00 (1.00–4.20) ^c^
Mohseni-Bandpei et al. 2007 [27]	TV (continuous)	Bivariate logistic regression	OR 0.66 (0.51–0.86)
	PC (continuous)	Bivariate logistic regression	OR 0.86 (0.58–1.28)
Skoffer and Foldspang 2008 [28]	TV or video (h/d), (continuous), weekend before research	Multivariate logistic regression	OR 1.07 (1.01–1.14) ^d^
	TV or video (h/d), (continuous), two days before research	Multivariate logistic regression	OR 1.18 (1.05–1.34) ^d^
Hakala et al. 2010 [29]	PC: <1 h/d, 1–3 h/d, ≥4 h/d vs. (vs. Not daily [Reference])	Multivariate logistic regression	OR <1 h/d: 0.8 (0.4–1.4); 1–3 h/d: 1.1 (0.7–1.7); ≥4 h/d: 2.6 (1.1–6.1) ^e^
Erne and Elfering 2011 [30]	TV, PC and video: <0.5 h/d, 0.5–1 h/d, 1–1.5 h/d, >1.5 h/d	Multivariate ordinal logistic regression	OR 0.48 (0.27–0.85) ^f^
Hakala et al. 2012 [32]	PC: 0.5–2 h/d, ≥2 h/d (vs. <0.5 h/d [Reference])	Multivariate logistic regression	OR Severe/Moderate LBP: 0.5–2 h/d: 1.60 (0.70–3.80); ≥2 h/d: 3.50 (1.50–8.30) ^g^
		Multivariate logistic regression	OR Mild LBP: 0.5–2 h/d: 2.40 (1.20–4.80); ≥2 h/d: 3.10 (1.50–6.70) ^g^
Graup et al. 2014 [33]	TV, PC and video games: >0.43 h/d vs. ≤0.43 h/d (Reference)	N/A	OR 1.17 (0.87–1.58) *
Minghelli et al. 2014 [34]	TV: >1.4 h/d vs. <1.4 h/d (Reference)	N/A	OR 1.09 (0.80–1.47) *
	PC and video games: >1.4 h/d vs. <1.4 h/d (Reference)	N/A	OR 0.90 (0,63–1.31) *
Fernandes et al. 2015 [35]	TV: >2 h/d vs. <2 h/d (Reference)	N/A	OR 2 h/d: 1.27 (0.92–1.76) *
	PC: >2 h/d vs. <2 h/d (Reference)	N/A	OR 2 h/d: 0.89 (0.64–1.24) *
Rossi et al. 2016 [36]	TV, PC, smartphone, video games, and tablet (h/d) (continuous)	Multivariate logistic regression	OR Boys: 1.07 (1.01–1.12) ^h^
			OR Girls: 1.06 (1.01–1.10) ^h^
Dianat et al. 2017 [37]	TV: 0.43–1.71 h/d, >1.71 h/d (vs. <0.43 h/d [Reference])	Bivariate logistic regression	OR 0.43–1.71 h/d: 1.02 (0.79–1.31); >1.71 h/d: 1.17 (0.91–1.50)
	PC: 0.14–0.57 h/d, >0.57 h/d (vs. <0.14 h/d [Reference])	Bivariate logistic regression	OR 0.14–0.57 h/d: 1.05 (0.83–1.35); >0.57 h/d: 1.03 (0.8–1.34)
	Video games: 0.14–0.28 h/d, >0.28 h/d (vs. <0.14 h/d [Reference])	Bivariate logistic regression	OR 0.14–0.28 h/d: 1.20 (0.92–1.55); >0.28 h/d: 0.96 (0.75–1.22)
Yabe et al. 2018 [38]	TV: 1–<2 h/d, 2–<3 h/d, ≥3 h/d (vs. <1 h/d [Reference])	Multivariate logistic regression	OR 1–<2 h/d: 0.91 (0.59–1.41); 2–<3 h/d: 0.84 (0.54–1.29); ≥3 h/d: 1.00 (0.66–1.51) ^i^
	Video games: 1–<2 h/d, 2–<3 h/d, ≥3 h/d (vs. <1 h/d [Reference])	Multivariate logistic regression	OR 1–<2 h/d: 1.36 (1.01–1.84); 2–<3 h/d: 1.46 (1.01–2.10); ≥3 h/d: 2.18 (1.49–3.20) ^j^
Ben Ayed et al. 2019 [39]	TV: 0.43–1.71 h/d, >1.71 h/d (vs. <0.43 h/d [Reference])	Multivariate logistic regression	OR 0.43–1.71 h/d: 1.00 (0.80–1.40); >1.71 h/d: 1.50 (1.10–2.10) ^k^
	PC: 0.14–0.57 h/d, >0.57 h/d (vs. <0.14 h/d [Reference])	Multivariate logistic regression	OR 0.14–0.57 h/d: 1.10 (0.80–1.43); >0.57 h/d: 1.56 (1.17–2.10) ^k^
	Video games: 0.14–0.28 h/d, >0.28 h/d (vs. <0.14 h/d [Reference])	Multivariate logistic regression	OR 0.14–0.28 h/d: 1.16 (0.83–1.60); >0.28 h/d: 1.83 (1.34–2.50) ^k^
Bento et al. 2020 [40]	TV: ≥3 h/d vs. <2 h/d (Reference)	Multivariate logistic regression	PR 1.17 (1.01–1.36) ^l^
	PC: ≥3 h/d vs. <2 h/d (Reference)	Bivariate logistic regression	PR 1.02 (0.91–1.14)
	Smartphone: ≥3 h/d vs. <2 h/d (Reference)	Multivariate logistic regression	PR 1.36 (1.11–1.68) ^l^
	Tablet: ≥3 h/d vs. <2 h/d (Reference)	Multivariate logistic regression	PR 1.46 (1.21–1.76) ^l^
Buabbas et al. 2020 [41]	Smartphone and tablet: 2–4 h/d, >4 h/d (vs. <2 h/d [Reference])	N/A	OR 2–4 h/d: 1.50 (1.05–2.16); >4 h/d: 2.40 (1.75–3.31) *
Minghelli 2020 [42]	TV: <1.4 h/d vs. >1.4 h/d (Reference)	Bivariate logistic regression	OR 1.09 (0.60–1.98)
	Smartphone: <1.4 h/d vs. >1.4 h/d (Reference)	Multivariate logistic regression	OR 2.39 (1.41–4.08) ^m^
	Video games: <1.4 h/d vs. >1.4 h/d (Reference)	Bivariate logistic regression	OR 1.13 (0.59–2.14)
Rezapur-Shahkolai et al. 2020 [43]	TV: 0.14–0.43 h/d, >0.43 h/d (vs. <0.14 h/d [Reference])	Penalized logistic regression	OR 0.14–0.43 h/d: 0.85 (0.50–1.44); >0.43 h/d: 2.62 (1.46–4.68) ^n^
de Vitta et al. 2021 [45]	TV: ≥3 h/d vs. <3 h/d (Reference)	Bivariate logistic regression	OR 0.90 (0.65–1.25)
	PC: ≥3 h/d vs. <3 h/d (Reference)	Bivariate logistic regression	OR 1.06 (0.75–1.49)
	Smartphone: ≥3 h/d vs. <3 h/d (Reference)	Multivariate logistic regression	OR 1.49 (1.11–2.00) ^o^
	Tablet: ≥3 h/d vs. <3 h/d (Reference)	Multivariate logistic regression	OR 3.21 (1.41–7.30) ^o^
Joergensen et al. 2021 [46]	TV, PC and video games: 2–<4 h/d, 4–<6 h/d, ≥6 h/d vs. <2 h/d [Reference])	Multivariate logistic regression	RR Boys (moderate LBP): 2–<4 h/d: 1.09 (0.95–1.25), 4–<6 h/d: 1.05 (0.90–1.22), ≥6 h/d: 1.22 (1.02–1.46) ^p^
			RR Boys (severe LBP): 2–<4 h/d: 1.02 (0.79–1.32), 4–<6 h/d: 1.16 (0.88–1.53), ≥6 h/d: 1.42 (1.03–1.94) ^p^
			RR Girls (moderate LBP): 2–<4 h/d: 1.17 (1.06–1.29), 4–<6 h/d: 1.30 (1.15–1.47), ≥6 h/d: 1.50 (1.26–1.78) ^p^
			RR Girls (severe LBP): 2–<4 h/d: 1.19 (1.01–1.39, 4–<6 h/d: 1.39 (1.15–1.69), ≥6 h/d: 2.64 (2.10–3.31) ^p^
da Costa et al. 2022 [47]	TV, PC, smartphone, and video games: 3.26–7.59 h/d, ≥7.60 h/d vs. <3.25 h/d [Reference])	Multivariate logistic regression	OR Boys: 3.26–7.59 h/d: 1.86 (0.92–3.77); ≥7.60 h/d: 1.71 (0.80–3.65) ^q^
			OR Girls: 3.26–7.59 h/d: 2.73 (1.45–5.02; ≥7.60 h/d: 2.49 (1.30–4.76) ^q^
Lemes et al. 2022 [48]	Smartphone: >2 h/d vs. <2 h/d (Reference)	Multivariate logistic regression	OR > 2 h/d: 1.42 (0.79–2.55) ^r^
van den Heuvel et al. 2023 [49]	TV (h/d) (continuous)	Bivariate logistic regression	OR 1.26 (0.97–1.64)
	Video games (h/d) (continuous)	Bivariate logistic regression	OR 0.92 (0.78–1.08)
Obeidat and AL-Shalabi 2025 [50]	Smartphone: >3 h/d vs. <3 h/d (Reference)	Bivariate logistic regression	OR > 3 h/d: 1.86 (1.34–2.60)

h/d: hours per day; TV: television; N/A: not applicable; PC: personal computer (desktop and laptop); OR: odds ratio; PR: prevalence ratio; RR: relative risk; RULA: Rapid Upper Limb Assessment; LBP: low back pain, Notes: ^a^. Adjusted for age, sex parent’s history of LBP, and competitive sport practice. ^b^. Adjusted for gender, distances walked or bicycled to school and other activities, and mutually adjusted for television/computer time and physical activity. ^c^. Adjusted for age, sex, school success, pubertal age, and stress symptoms. ^d^. Adjusted for age and sex, body mass index, physical activity and inactivity indicators, weight and transport of the schoolbag, school, smoking, school furniture, home furniture, playing handball, playing football, jogging, performing gymnastics, doing homework, and reading. ^e^. Adjusted for age, sex and parents’ education. ^f^. Adjusted for age, sex, flexibility, BMI, backpack weight, self-reported sports activities, back pain reported by the mother, back pain reported by the father, psychosocial stress, report of poor grades in mathematics, and recommendation for type of secondary school. ^g^. Adjusted for sex and school grade. ^h^. Adjusted for age, BMI, chronic diseases, smoking, and school success. ^i^. Adjusted for gender, age, BMI, type of sport activity (recreational, local competitive, etc.), frequency of sport practice (days per week), duration of sport practice (hours per week), daily time spent playing video games. ^j^. Adjusted for gender, age, BMI, type of sport activity (recreational, local competitive, etc.), frequency of sport practice (days per week), duration of sport practice (hours per week), daily TV time. ^k^. Adjusted for age, BMI, school grade in primary school, school grade in high school, residence (rural/urban), socioeconomic status, father’s education, weekly hours of sports, daily hours of computer use, daily hours of video games, seat backrest height position, seat width position, supplementary tutoring, schoolbag weight as a percentage of body weight, time spent carrying the schoolbag, and method of schoolbag carriage (both shoulders, one shoulder, by hand). ^l^. Adjusted for gender, PC type (desktop or laptop), daily smartphone use, posture while using the smartphone, and tablet use (yes or no). ^m^. Adjusted for age and sitting posture. ^n^. Adjusted for age (≥10 years), seat backrest height (very low), backrest curvature (very curved), desk height (very low), family history of LBP, schoolbag carriage method (one shoulder), RULA score (levels 3 and 4), time spent carrying schoolbag >20 min/day. ^o^. Adjusted for sex, mental health, and mutually adjusted for other screen types. ^p^. Adjusted for age, parity, family type, parental education, household income, and simultaneously modeled the interaction between screen time, sex, and physical activity behavior. ^q^. Adjusted for socioeconomic status, physical activity and waist circumference. ^r^. Adjusted for age, sex, BMI, abdominal obesity, socioeconomic status and sleep quality. * OR and 95% CI calculated from raw data reported in the article. Note: When both crude and adjusted models were reported, the adjusted model was preferred. If multiple adjusted models were available, we selected the one that included the most relevant covariates based on our judgment.

## Data Availability

No new data were created or analyzed in this study. Data sharing is not applicable to this article.

## Supplementary Materials

The following supporting information can be downloaded at https://www.mdpi.com/article/10.3390/healthcare14020233/s1. Table S1. PRISMA Checklist; Table S2. Search strategy for Meta-analysis; Table S3. Coded variables.

## Abbreviations

The following abbreviation is used in this manuscript:

LBP	Low back pain
