# Supplementary material for: Sedentary Behavior and Low Back Pain in Children and Adolescents: A Systematic Review and Meta-Analysis
Source: Healthcare (Basel). 2026 Jan 16;14(2):233. doi: 10.3390/healthcare14020233 (PMC12841405; doi:10.3390/healthcare14020233)
Supplement: Supplementary file 1 [file healthcare-14-00233-s001.zip › Supplementary Table S2. Search strategy.pdf]

Table S2. Search strategy

Database	Search strategy	Number of results
**Pubmed**	("low back pain" OR "back pain" OR backache OR LBP) AND (sedent* OR "sedentary behavior" OR "screen time" OR "sitting time" OR TV* OR computer* OR mobile phone* OR videogame* OR "video game*") AND (prevalence OR "risk factor*" OR associat* OR inciden*) AND (child* OR adolescen* OR teen* OR youth OR school*)	618
**Scopus**	("low back pain" OR "back pain" OR backache OR LBP) AND (sedent* OR "sedentary behavior" OR "screen time" OR "sitting time" OR TV* OR computer* OR mobile phone* OR videogame* OR "video game*") AND (prevalence OR "risk factor*" OR associat* OR inciden*) AND (child* OR adolescen* OR teen* OR youth OR school*)	2715
**Web of Science (WOS)**	("low back pain" OR "back pain" OR backache OR LBP) AND (sedent OR "sedentary behavior" OR "screen time" OR "sitting time" OR TV OR computer OR mobile phone OR videogame OR "video game") AND (prevalence OR "risk factor" OR associat OR inciden) AND (child OR adolescen OR teen OR youth OR school)	282
**Scielo**	("low back pain" OR "back pain" OR backache OR LBP) AND (sedent* OR "sedentary behavior" OR "screen time" OR "sitting time" OR TV* OR computer* OR mobile phone* OR videogame* OR "video game*") AND (prevalence OR "risk factor*" OR associat* OR inciden*) AND (child* OR adolescen* OR teen* OR youth OR school*)	18
**PEDro**	"sedentary behavior" children<br><br>Advanced search: Match all search terms (AND)<br><br>Adolescent* low back pain	22
**LILACS**	("low back pain" OR "back pain" OR backache OR LBP) AND (sedent* OR "sedentary behavior" OR "screen time" OR "sitting time" OR TV* OR computer* OR mobile phone* OR videogame* OR	29

	"video game*") AND (prevalence OR "risk factor*" OR associat* OR inciden*) AND (child* OR adolescen* OR teen* OR youth OR school*)	
**PsycINFO**	("low back pain" OR "back pain" OR backache OR LBP) AND (sedent* OR "sedentary behavior" OR "screen time" OR "sitting time" OR TV* OR computer* OR mobile phone* OR videogame* OR "video game*") AND (prevalence OR "risk factor*" OR associat* OR inciden*) AND (child* OR adolescen* OR teen* OR youth OR school*)	83
**ScienceDirect**	("low back pain" OR "back pain") AND ("sedentary behavior" OR "screen time") AND (prevalence OR association) AND (children OR adolescents)	344
**CochraneLibrary**	("low back pain" OR "back pain" OR backache OR LBP) AND (sedent* OR "sedentary behavior" OR "screen time" OR "sitting time" OR TV* OR computer* OR mobile phone* OR videogame* OR "video game*") AND (prevalence OR "risk factor*" OR associat* OR inciden*) AND (child* OR adolescen* OR teen* OR youth OR school*)	59
**Total**		4170
