# Supplementary material for: Sedentary Behavior and Low Back Pain in Children and Adolescents: A Systematic Review and Meta-Analysis
Source: Healthcare (Basel). 2026 Jan 16;14(2):233. doi: 10.3390/healthcare14020233 (PMC12841405; doi:10.3390/healthcare14020233)
Supplement: Supplementary file 1 [file healthcare-14-00233-s001.zip › Supplementary Table S3. Coded variables.pdf]

Table S3. Coded variables

Type of variables	Coded variables
**Context**	(a) continent; (b) country.
**Participant characteristics**	(a) source of the participants (where the sample was collected: school field, sports field, health field, community, or others); (b) number of participants in each category according to daily screen time (hours per day); (c) number of participants with and without LBP; (d) type of association measure used (odds ratio, relative risk, correlation coefficients, or prevalence estimates); (e) age (mean and SD, in years), (f) age category (children, adolescent or mixed); (g) sex of the sample (percentage of females); (h) definition of sedentary lifestyle or sedentary behavior (World Health Organization, etc.); (i) percentage of participants practicing recreational sport; (j) percentage of participants practicing competitive sport; (k) percentage of participants practicing contact sport; (l) physical activity level of subjects (times a month), (m) physical activity level of subjects (hours a week); (n) daily screen time (hours per day), stratified according to the presence or absence of LBP.
**Methodological**	(a) study design (cohort, case-control, cross-sectional); (b) data collection method (questionnaire, interview, or parental/guardian report); (c) prevalence type (lifetime, point, or period in months).
**Extrinsic**	(a) year the study was conducted.
